# Maize chromatin introgression’s effect in oat × maize addition lines

**DOI:** 10.1007/s13353-026-01086-3

**Published:** 2026-06-18

**Authors:** Katarzyna Juzoń-Sikora, Anna Nowicka, Mahmoud Said, Dominika Idziak-Helmcke, Tomasz Warzecha, Marzena Warchoł, Edyta Skrzypek, Ales Pecinka

**Affiliations:** 1 The F. Górski Institute of Plant Physiology, Polish Academy of Sciences, Kraków, Poland; 2 Centre of Plant Structural and Functional Genomics, Institute of Experimental Botany of the Czech Academy of Sciences, Olomouc, Czech Republic; 3 Field Crops Research Institute, Agricultural Research Centre, Giza, Egypt; 4 Institute of Biology, Biotechnology, and Environmental Protection, University of Silesia in Katowice, Katowice, Poland; 5 Department of Plant Breeding, Physiology, and Seed Science, University of Agriculture in Kraków, Kraków, Poland

**Keywords:** Oat × maize addition lines, Rabl configuration, Chromatin introgression, Genomic stability

## Abstract

The addition lines derived from oat (*Avena sativa* L.) × maize (*Zea mays* L.) crosses constitute a unique tool for studying gene expression and the structure of chromatin, as well as a source of agronomically desirable traits in the context of new intergeneric hybrid cultivars. Therefore, for a better understanding of the introgression of maize chromatin into the oat genome, a comprehensive analysis of oat × maize lines was conducted. The presence of maize chromatin was confirmed in three tested lines by PCR-based amplification of the maize retrotransposon *Grande-1*, and verified by genomic in situ hybridization. Interestingly, the addition lines containing cytologically-detectable maize chromosomes showed a possible disturbance of the Rabl-like centromere-telomere polarization typical for oat. Moreover, the maize chromatin was consistently positioned near the nuclear periphery. The maize chromatin introgression caused a prolonged DNA replication phase, altering cell cycle dynamics, and may led to mitotic abnormalities, such as the formation of micronuclei and anaphase chromosome bridges. The introgression lines also showed morphological and physiological changes: smaller seeds, hampered seedlings’ growth, and reduced yield parameters (number of flowers/seeds per plant/panicle and seed weight). These findings shed more light on the consequences of the maize chromatin introgression into the oat genome and the stability and utility of the obtained hybrids.

**Supplementary Information:**

The online version contains supplementary material available at https://doi.org/10.1007/s13353-026-01086-3.

## Introduction

The first successful interspecific cross was performed in barley by Kuckuck (1934) between *Hordeum vulgare* (cultivated barley) and *Hordeum bulbosum* (bulbous barley grass) resulting in one sterile triploid hybrid plant. Notably, in this hybrid, chromosome elimination did not occur. Later, Symko (1969), Kasha and Kao (1970), and Lange (1971) proposed the hypothesis of chromosome elimination as a mechanism of haploid barley production. This was ultimately confirmed by Kasha and Kao (1970) after hybridizations of diploid *H. vulgare* with diploid *H. bulbosum*, which resulted in regeneration of haploid *H. vulgare* plants through complete loss of the *H. bulbosum* genome after rescuing embryos into a dedicated nutrient medium. Nowadays, the ‘’bulbosum method’’ has been superseded by the anther and microspore cultures, as these methods are more efficiently used in regenerating doubled haploids (DH) (Houben et al. 2011). Intergeneric crossings (alias wide crossings) are frequently used to generate DH plants in breeding initiatives (Ferrie et al. 2014; Houben et al. 2011; Skrzypek et al. 2018). This procedure involves chromosomal elimination from one of the parents, resulting in homozygosity in the first generation, which is advantageous for breeders because it reduces the time required to fix the genetic constitution and thus develop new cultivars with a uniform phenotype.

Such interspecific crosses trigger a genome conflict in the nuclei of the newly formed hybrid (Thondehaalmath et al. 2021). The result of interspecific crosses can be: (i) the uniparental genome elimination resulting in the haploid embryos formation after successful fertilization (reviewed in Ishii et al. 2016); (ii) the loss of a few chromosomes, which leads to aneuploid offspring, e.g. *Brassica* (Kang et al. 2014), cotton (Chen et al. 2014; Wang et al. 2016), grasses: *Hordeum lechleri* × *H. vulgare*, *Triticum aestivum* × *H. vulgare* (Barclay 1975), or *Avena sativa* × *Zea mays* (Ananiev and Riera-Lizarazu 1997; Kynast et al. 2001; Riera-Lizarazu et al. 1996); (iii) both parental genomes coexist as interspecific hybrid, e.g. *Triticale* (Zillinsky 1974) and *Apriplum* (Okie 2005). The complete elimination of one of the parental genomes mentioned above is not uncommon: 74 hybrids involving monocotyledonous (0.12%) and 35 involving dicotyledonous (0.02%) species have been reported to be subjected to uniparental chromosome elimination (Ishii et al. 2016).

Chromosome elimination leads to genetic and epigenetic changes, chromosomal structural rearrangements, and deletions (Gernand et al. 2005; Jones and Hegarty 2009). This process is known to be dependent on genetic factors, temperature after fertilization, and genome balance between the parental species (Kalinowska et al. 2019). The mechanisms involved in uniparental chromosome elimination during hybrid embryo development include differences in the timing of essential mitotic processes, asynchrony in the synthesis of different subunits of nucleoprotein complexes of chromatin, spatial separation of genomes during interphase (Linde-Laursen and von Bothmer 1999), parent-specific inactivation of centromeres (Mochida et al. 2004), as well as degradation of alien chromosomes by the host-specific nuclease activity (Gernand et al. 2005).

While much attention has been given to interspecific crossings, such as those in *Hordeum*, intergeneric crossings between oat and maize also plays a crucial role in plant genetics. In the crosses between oat and maize some of the hybrid embryos developed into aneuploid plants with a complete set of oat chromosomes and a different number of maize chromosomes added. Similarly, Ishii et al. (2013) reported that in crosses of oat and pearl millet (*Pennisetum glaucum* L.), chromosome and marker analyses showed that the seedlings were hybrids possessing an entire oat chromosome complement and only some of the pearl millet chromosomes. However, obtained hybrids showed only shoot growth, which finally died under the given light regime.

The partial somatic elimination of maize chromosomes occurs in oat × maize addition (OMA) lines resulting from oat (*Avena sativa* L., 2 *n* = 6x = 42, A_s_A_s_C_s_C_s_D_s_D_s_) × maize (*Zea mays* L., 2 *n* = 2x = 20, Zm, genome = MM) wide crossing. In OMA lines, the elimination of maize chromosomes happens due to the failure of spindle fiber attachment to the centromeres, which limits their mobility for the duration of mitotic metaphase and anaphase (Laurie and Bennett 1989). Kynast et al. (2001) showed that in OMA lines the preserved maize chromosomes were stable and acted as oat chromosomes in mitosis. What is more, amongst maize crosses with other cereals, only OMA lines formed embryos that were able to germinate and grow into partially fertile plant. This is possible because of meiotic restitution, which improves the production of unreduced gametes and allows for the transmission of the added maize chromosomes (Kynast et al. 2001).

The OMA lines constitute a valuable research material for analyses of e.g. maize knob and centromere structure (Ananiev et al. 1998), meiotic chromosome behaviour (Bass et al. 2000), and physical mapping of single-copy sequences on maize chromosomes by fluorescence in situ hybridization (FISH) (Amarillo et al. 2007). There are indications that OMA lines, as hybrids between C3 and C4 plants, may predominate oat cultivars in terms of functioning of the photosynthetic apparatus (Warchoł et al. 2024), both in optimal and stress conditions (Juzoń et al. 2020). Warchoł et al. (2024) showed that maize chromosomes in the oat genome can modify the size of the bundle sheath cells, the trait associated with C4 photosynthesis, depending on the oat genotype. They also observed changes in flag leaf width, height of main shoot, panicle length, and weight of a thousand grains per plant. Juzoń et al. (2020) showed that, compared to oat *cv*. Bingo, OMA lines were characterized by higher values of most of the analyzed chlorophyll *a* fluorescence (CF) parameters (Area, PI, RC/CS_m_), as well as pigment content (Chl_a_, Chl_b_, Car). Moreover, they postulated that the functioning of the photosynthetic apparatus in OMA lines depends not only on the number of preserved maize chromosomes but on their interaction with a given oat genotype.

Improvements in photosynthesis and the ascension of photosynthetic utilization can be an effective way to increase the yield potential (Wang et al. 2015). OMA lines also constitute a source of valuable variation in plant breeding while generating new hybrid cultivars and may help in understand plant susceptibility to unfavorable environmental condition, e.g. drought. However, for this to happen, the acquired features must be maintained in subsequent generations.

Therefore, the objective of this work was to estimate the obtained hybrids in the context of their usefulness in breeding of new cultivars that are more insusceptible to biotic and abiotic stress factors. We performed a comprehensive analysis of five oat × maize lines to investigate the impact of maize chromatin introgression on nuclear organization, cell cycle dynamics, genomic stability, and reproductive development. Using both molecular and cytogenetic methods, we characterized the maize chromatin introgression and its possible effects on the Rabl-like configuration of oat nuclei. We also presented the mitotic abnormalities, changes in cell cycle, and key phenotypic traits related to panicle and seed development. Our findings provide new insights into the interaction between alien chromatin and the host genome, as well as the probable effect of alien chromatin on plant development and breeding.

## Materials and methods

### Plant material and growth conditions

The plant material used in this study included five F3 OMA lines. OMA lines were obtained by wide crossing of oat (*A. sativa* L., 2 *n* = 6x = 42) with maize (*Z. mays* L. var. *saccharata*, 2 *n* = 2x = 20) *cv*. Waza followed by colchicine treatment. For the production of OMA lines, five oat genotypes: DC 06011-6 × POB 722, STH 9511 × Bingo, STH 8–50 × Canyon, STH 9110 × Contender, STH 93 − 61 × DC 2112/07 obtained from Plant Breeding Strzelce Ltd., PBAI Group, Strzelce, Łódź Voivodeship, and Małopolska Plant Breeding Ltd. HBP Polanowice, Poland, were pollinated with maize. The wide crosses carried out at The *Franciszek Górski* Institute of Plant Physiology, Polish Academy of Sciences, Kraków, Poland generated following F1 OMA lines: line 18; 2n = 46 (42 + Zm1′′ + Zm2′′), line 23; 2n = 44 (42 + Zm2′′), line 55; 2n = 43 (42 + Zm3′), line 81; 2n = 44 (42 + Zm5′′), line 95; 2n = 42 (42 + Zm6). Oat *cv*. Bingo and maize *cv*. Waza were used as control plants. The presence of maize chromatin introgressions in F2 generation of OMA plants had been confirmed by PCR with primers that specifically amplify a maize retrotransposon *Grande-1* (Idziak-Helmcke et al. 2020; Skrzypek et al. 2018). The specific maize chromosomes present in the OMA lines were identified using SSR markers specific to individual maize chromosomes (Idziak-Helmcke et al. 2020; Juzoń et al. 2020).

For germination, ten seeds per genotype were placed on moist filter paper in Petri dishes, stratified at 4 °C in darkness for 48 h, and then incubated at 25 °C in the dark for 3 days. The germinated seedlings were transferred to Magenta boxes containing perlite saturated with Hoagland nutrient solution and grown under long-day conditions (16 h light/8 h dark; 20 °C day/16°C night; 60% relative humidity) in a greenhouse. After two weeks, seedlings were transplanted into 15 × 15 × 15 cm pots filled with a 2:1 (v/v) mixture of soil and sand. Plants were maintained under the same greenhouse conditions until flowering.

### DNA isolation and molecular identification of maize chromatin in OMA lines

Identification of hybrids was carried out using the PCR method, with the application of the marker retrotransposon fragment sequence *Grande-1*, which is found across all maize chromosomes (Li et al. 2001).

Genomic DNA was isolated using the DNeasy Plant Mini Kit (Qiagen, Hilden, Germany), according to the manufacturer’s protocol. Next, dried DNA samples were diluted in 1 × TE buffer (Tris, EDTA) for 48 h. The concentration of the DNA solution was measured at 260 nm with the NanoDrop 2000c spectrophotometer (Thermo Scientific, USA). Finally, the DNA concentration of 50 ng/µl was used in the PCR reaction. The maize DNA isolated from *cv*. Waza was used as a positive control, and oat DNA from *cv*. Bingo as a negative one. PCR reactions were performed using the JumpStart™ REDTaq^®^ ReadyMix™ (Sigma-Aldrich, Merck KGaA, Darmstadt, Germany) with primers specific to *Grande-1*: *Grande-1F* (5’ - AAAGACCTCACGAAAGGCCCAAGG − 3’) and *Grande-1R* (5’ - AAATGGTTCATGCCGATTGCACG − 3’) (GenBank, accession number X97604). The PCR reaction was carried out under the following conditions: 1 × (94 °C for 5 min), 25 × (94 °C for 30 s → 58 °C for 30 s → 72 °C for 30 s), (72 °C for 5 min → 4 °C). The analysis of the obtained PCR products after electrophoretic separations was performed using the GelScan version 1.45 (Kucharczyk - Electrophoretic Techniques, Poland).

### Mitotic chromosome preparations

Mitotic metaphase chromosomes of the synchronized root tips of F3 OMA lines were prepared following the protocol of Vrána et al. (2016) and Doležel et al. (2023) with minor modifications. The OMA seeds, oat *cv*. Bingo and maize *cv*. Waza were germinated for 3–4 days in the dark on moist filter paper in Petri dishes. The seedlings were transferred to Hoagland’s nutrient solution supplemented with 2 mM hydroxyurea to synchronize the cell cycle. To accumulate cells in metaphase, the roots were treated for 2 h in Hoagland’s solution containing 2.5 µM amiprophos-methyl (APM, A0185, Duchefa Biochemie, The Netherlands). After treatment, root tips were fixed in ice-cold 90% acetic acid for 10 min, washed three times in 70% ethanol, and stored at − 20 °C in 70% ethanol until use. Then, root tips were excised and washed in 0.01 M citric acid/sodium citrate buffer (pH 4.8) for 15–20 min. The chromosomes were prepared using the drop technique described by Danilova et al. (2012). Root tips were enzymatically macerated at 37 °C for 90 min in a digestion mixture containing 4% (w/v) cellulase Onozuka R-10 (150422-01, Yakult Pharmaceutical Industry, Japan) and 1% (w/v) pectolyase Y23 (9033-35-6, Duchefa, Biochemie, The Netherlands) in KCl buffer (75 mM KCl, 7.5 mM EDTA, pH 4.0). When digestion was completed, digested root-tip samples were treated in ice-cold 400 µl 1× TE buffer (Tris-EDTA buffer, pH 7.6) followed by 3× washing in ice cold 100% ethanol. Finally, 7 µl ice-cold acetic acid: methanol mix (9:1) were added, the root tips were smashed, and suspension was dropped from 5 cm height onto glass slides, placed in a humid box until desiccation. One hundred and twenty crushed preparations were made from root apical meristems. The quality of chromosome spreads was assessed using a phase-contrast microscope (Primo Star, Zeiss, Germany), and best slides containing at least five clear metaphase spreads for each plant were selected for genomic in situ hybridization (GISH).

### Genomic in situ hybridization

To distinguish maize chromosomes in the oat background the total genomic DNA (gDNA) of maize *cv*. Waza was used. Maize gDNA was extracted using the Nucleon PhytoPure genomic DNA Preparation Kit (GE – Healthcare, USA).

The DNA was labeled with biotin using the BIOT-Nick Translation Kit (Roche Applied Science, Germany) following the manufacturer’s instructions. As an additional chromosome marker, a 35 S rDNA (ribosomal DNA) probe was prepared from the plasmid pTa71, which contains a 9.1 kb fragment of the 35 S rDNA repeat from bread wheat (Gerlach and Bedbrook, 1979). The 35 S rDNA probe was labeled with digoxigenin using the DIG-Nick Translation Kit (Roche Applied Science, Germany).

GISH was performed according to Nowicka et al. (2023) with slight modifications. Chromosome preparations were air-dried at room temperature (RT), rinsed in 1× PBS, post-fixed in 4% formaldehyde in 1× PBS for 15 min, then rinsed again with 1× PBS. The hybridization mixture contained 50 ng µl⁻¹ of biotin-labeled maize gDNA, 40 ng µl⁻¹ of digoxigenin-labeled 35 S rDNA, 50% (v/v) deionized formamide, 10% (v/v) dextran sulfate, and 2× SSC. The mixture was denatured at 95 °C for 4 min, cooled on ice, and then co-denatured with chromosomal DNA on slides at 80 °C for 3 min. Biotin-labeled probe was detected with streptavidin-Cy3 (1:500; Molecular Probes, SA1010), while digoxigenin-labeled probe was visualized using anti-digoxigenin antibodies conjugated with fluorescein isothiocyanate (FITC, 1:200; 11207741910; Roche, Germany). Chromosomes were counterstained with 1.5 µg ml^− 1^ 4’,6-diamidino-2-phenylindole (DAPI) in Vectashield antifade mounting medium (H-1200-10,Vector Laboratories, US).

### Isolation of nuclei and flow sorting

Nuclei were isolated from root apical meristems (RAMs) of F3 OMA lines as described by Nowicka et al. (2023) with minor modifications. For each sample, approximately 40 roots were collected from seedlings at 2 days post-germination. Roots were cut ~ 1 cm from the tip and placed in a drop of distilled water. After briefly draining on cellulose tissue paper, roots were rinsed in 10 mM Tris buffer (pH 7.0), fixed in 2% (v/v) formaldehyde in Tris buffer for 20 min on ice, and then washed three times for 5 min each in fresh, ice-cold Tris buffer. Root apices (~ 1 mm from the tip) were excised and homogenized in 500 µl of LB01 buffer (15 mM Tris–HCl pH 7.5, 2 mM Na₂EDTA, 0.5 mM spermine, 80 mM KCl, 20 mM NaCl, and 0.1% Triton X-100) using a Polytron PT1300D homogenizer (Kinematica AG, Switzerland) at 15,000 rpm for 13 s. Crude homogenates were sequentially filtered through 50 μm and 20 μm nylon mesh filters. The resulting nuclei suspensions were stained with 2 µg ml⁻¹ DAPI and analyzed by flow cytometry at rates of 1,000–2,000 particles per second. Bivariate flow karyotypes of DAPI pulse area (DAPI-A) versus DAPI pulse width (DAPI-W) fluorescence were acquired, and 20,000 events were recorded to create a bivariate flow karyotype for each experiment. Sort regions were set on the flow karyotypes, and G1 nuclei were flow sorted at rates of 20–50 per second. Approximately 500 nuclei corresponding to G1 phase were sorted into a 2 µl drop of sorting buffer (100 mM Tris–HCl pH 7.5, 50 mM KCl, 2 mM MgCl₂, 0.05% Tween-20, and 5% sucrose) on poly-L-lysine-coated microscope slides (Menzel Gläser, J2800AMNZ) using a FACSAria II SORP flow cytometer and sorter (BD Biosciences, San Jose, CA, USA). Slides were air-dried at RT for 1 h and stored at -20 °C until use.

### Immunofluorescence in situ hybridization (immuno-FISH)

In order to investigate the chromatin organization in the OMA nuclei, a combined immuno-FISH/GISH approach was applied using three cytogenetic markers: (i) a grass-specific anti-CENH3 antibody to detect centromeres in both oat and maize; (ii) oligonucleotide probe targeting the Arabidopsis-type telomeric repeat to visualize telomeres from both genomes; and (iii) maize gDNA to identify maize chromatin.

Slides were air-dried at RT, post-fixed in 4% formaldehyde in 1× PBS for 15 min, and washed with 1× PBS. Immunostaining was performed as described by Jasencakova et al. (2000) with minor modifications. Briefly, chromosome preparations were incubated overnight at 4 °C with a rabbit anti-grass CENH3 primary antibody (1:500; Nagaki et al. 2004), followed by incubation with a goat anti-rabbit secondary antibody conjugated to Alexa Fluor 488 (1:300; Molecular Probes, A11008) for 90 min at 37 °C. Before conducting FISH/GISH, slides were fixed in freshly prepared 3:1 ethanol: acetic acid for 10 min, followed by fixation in 3.7% formaldehyde in 1× PBS for another 10 min. Slides were then washed with 1 × PBS. Telomeres were detected using a synthetic 28-mer oligonucleotide probe [(5ʹ-CCCTAAA-3ʹ)₄], corresponding to the Arabidopsis-type telomeric repeat, labeled at the 5ʹ end with Cy5 (Eurofins). FISH/GISH procedures were performed as described in the previous section using a telomeric probe concentration of 40 ng µl⁻¹.

### Confocal microscopy

The images were taken with an AxioImager Z2 upright microscope (Zeiss, Germany) equipped with a pE-4000 LED illuminator light source (CoolLED, Andover, UK), motorized four-position excitation filter wheel, laser-free confocal spinning disk device (DSD2, Andor, Belfast, UK), and a ×100/1.4 NA Oil M27 Plan-Apochromat (Zeiss, Germany) objective. Images were acquired separately for each fluorochrome using the appropriate excitation [DAPI λ = 390/40 nm, green fluorescent protein (GFP) λ = 482/18 nm, red fluorescent protein (RFP) λ = 561/14 nm, Cy5 = 640/14 nm] and emission [DAPI λ = 452/45 nm, GFP λ = 525/45 nm, RFP λ = 609/54 nm, Cy5 = 676/29 nm] filters. For fluorescence detection, a 4.2 megapixel sCMOS camera (Zyla 4.2) was used and iQ 3.6.1 acquisition software (both Andor, Belfast, UK) was used to drive the microscope.

### Cell cycle and cell division analysis

For cell cycle analysis by flow cytometry, root tips from 2-day-old seedlings of F3 OMA lines were excised and finely chopped with a razor blade in 500 µl of Otto I solution (0.1 M citric acid, 0.5% Tween-20). The resulting suspension was filtered through a 50 μm nylon mesh (Sysmex-Partec GmbH, Germany) and nuclear DNA was stained by adding 1 ml of Otto II solution (0.4 M Na₂HPO₄·12 H₂O) containing 2 µg ml⁻¹ DAPI. Ten individual plants were analyzed per line. Measurements were performed using a CyFlow Space flow cytometer (Sysmex-Partec GmbH, Germany) equipped with a UV-LED excitation source and data were processed using FloMax software (Sysmex-Partec GmbH, Germany). In addition, mixed samples were prepared by simultaneously chopping one root from the *cv*. Bingo cultivar and one root from each individual OMA line in the same preparation to allow direct comparison of nuclear DNA content.

For the analysis of cell division abnormalities, roots from 2-day-old seedlings were washed in distilled water for 10 min, hydrolyzed in 5 N HCl at 60 °C for 5 min, stained with Feulgen solution for 30–40 min, and squashed in 45% acetic acid. Slides were mounted in Entellan (Merck, Germany) for microscopic observation. Images were captured using a light microscope (Olympus BX60, Olympus Corporation, Japan) equipped with a Promicra 3-3CP camera (Sony Corporation, Japan).

### Measurement of seeds, seedling, and panicles parameters

The seeds and seedlings of oat and OMA plants used in this study (F3) were documented by a Nikon D5600 digital camera equipped with an 80 mm Nikkor lens (both Nikon, USA). The following measurements were made: seed length [mm], seed area [mm^2^], root length [cm], coleoptile length [cm]. Seed parameters were measured using the ImageJ software (https://imagej.nih.gov/ij/), calibrated with an internal size reference. The measurements were performed on 10 seeds/plants per line to ensure biological replication. Additionally, the part of the F3 generation seeds was germinated, planted as described above in the subsection *2.1 Plant material and growth conditions*, and maintained under greenhouse conditions until flowering. Panicle morphology was documented using the Nikon D5600 with the same lens configuration. Seeds of the F3 generation were collected at the physiological maturity stage (GS95; BBCH-scale) and stored at RT. At least 5 plants from each line were analyzed to determine the number of flowers per plant/panicle, number of seeds per plant/panicle, and dry seeds weight, which was measured using an analytical scale (Sartorius, Germany).

### Statistical analysis

All measurement data were first tested for normality, followed by one-way ANOVA. Significant differences between means were determined using Duncan’s post hoc test at *p* < 0.05. Statistical analyses were performed using STATISTICA v. 13.1 (TIBCO Software Inc., Palo Alto, CA, USA).

## Results

### Molecular and cytogenetic characterization of maize chromatin introgressions in OMA lines

**Fig. 1:**
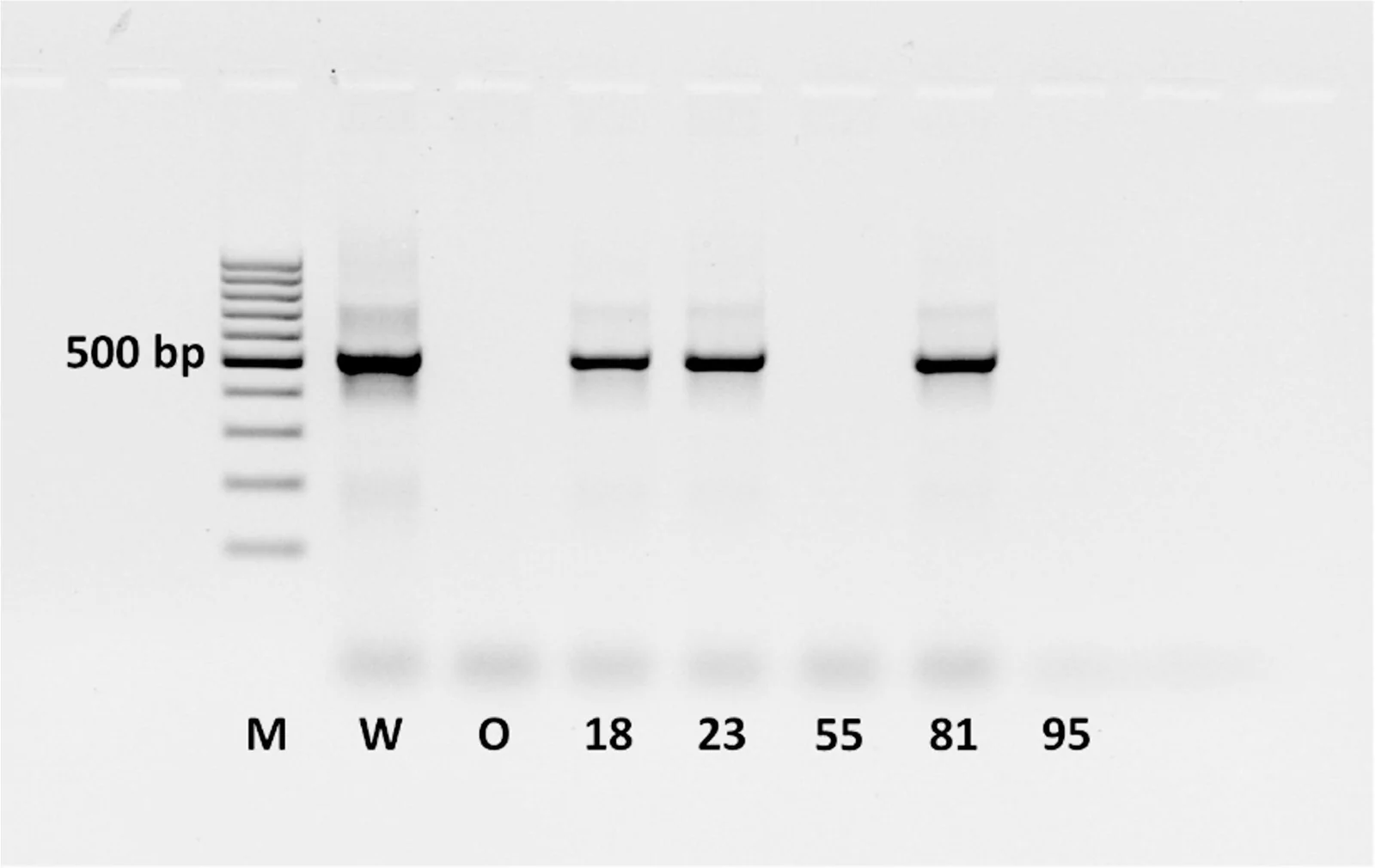
Electrophoretic separation of PCR products in 1.5% (w/v) agarose gel with the JumpStart RedTaq polymerase. Bands represent 500 bp DNA fragments that were amplified with marker *Grande-1*. The marker leader (M) is demonstrated in the first line. Maize *cv*. Waza (W) specificity is shown by product presence in maize DNA (positive control) and absence in oat *cv*. Bingo (O) DNA (negative control). The presence of retained maize chromosomes is indicated in 3 lines: 18 (42 + Zm1′′ + Zm2′′), 23 (42 + Zm2′′), and 81 (42 + Zm5′′) (OMA lines), while the lack of maize chromosomes is presented in 2 lines: 55 and 95. Photo credit: Tomasz Warzecha

Five OMA lines: 18, 23, 55, 81, and 95 were analyzed to assess the presence and extent of maize chromatin influence on the oat phenotype (Fig. 1). Introgression of maize genetic material was found in 3 out of 5 tested genotypes resulting from wide crossing of oat with maize. The presence of the maize retrotransposon fragment *Grande-1* (500 bp) was reconfirmed in the F3 generation of lines: 18, 23, and 81. The other two tested genotypes (lines: 55 and 95) did not show the maize genome elements in the F3 generation, even though their presence was found in the previous generations (Juzoń et al. 2020). These lines likely lost all the introgressed maize chromatin and now represent true DH lines.

**Fig. 2:**
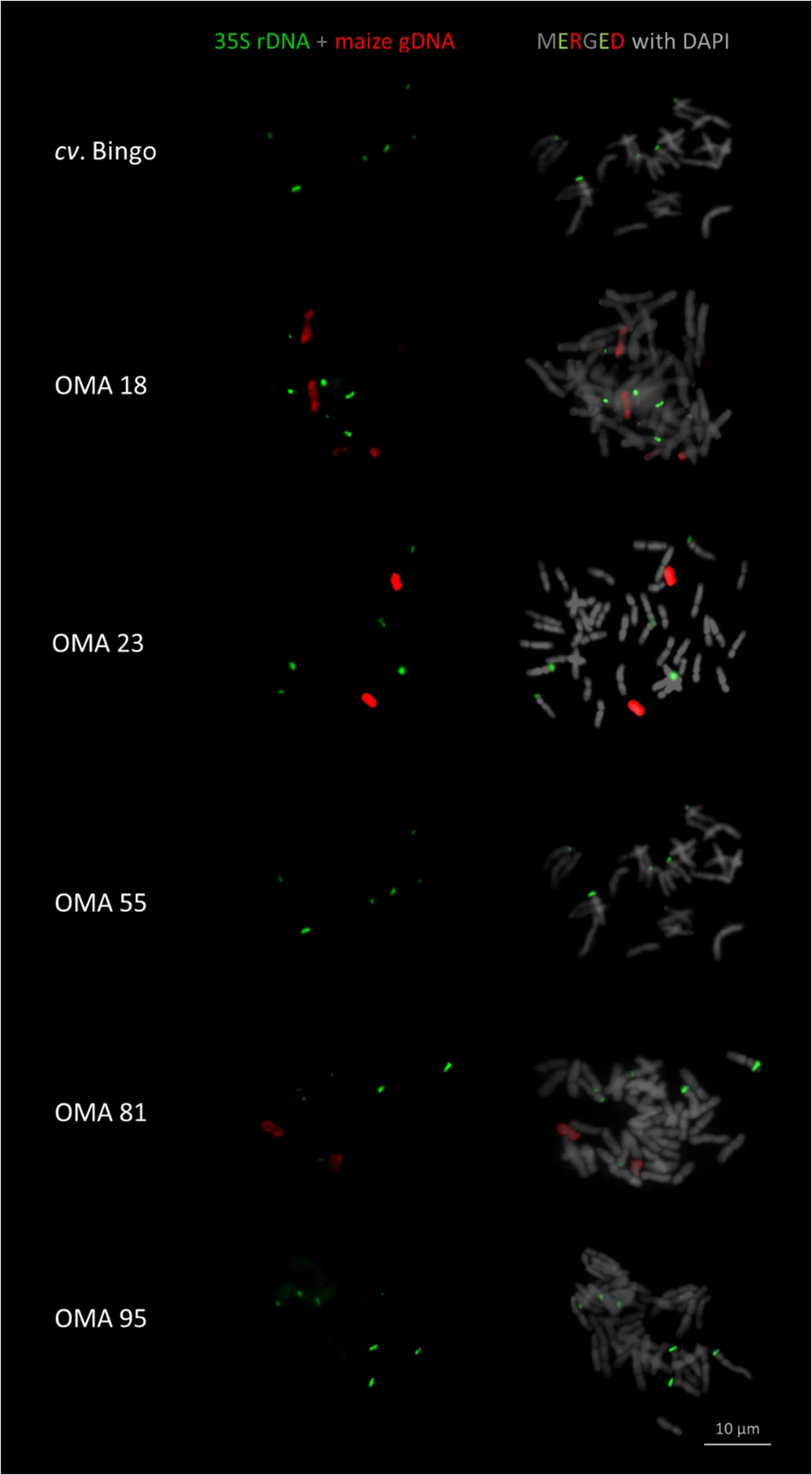
Identification of maize chromosome additions. Visualization of added maize chromosomes in oat genome by GISH: oat *cv*. Bingo, 2n = 42; line 18 (2n = 46 = 42 + Zm1′′ + Zm2′′); line 23 (2n = 44 = 42 + Zm2′′); line 55 (2n = 42); line 81 (2n = 44 = 42 + Zm5′′); line 95 (2n = 42). Red fluorescence: maize genomic DNA (gDNA); green fluorescence: 35 S rDNA; chromosomes counterstained with DAPI. Scale bar: 10 μm. Photo credit: Katarzyna Juzoń-Sikora

**Fig. 3:**
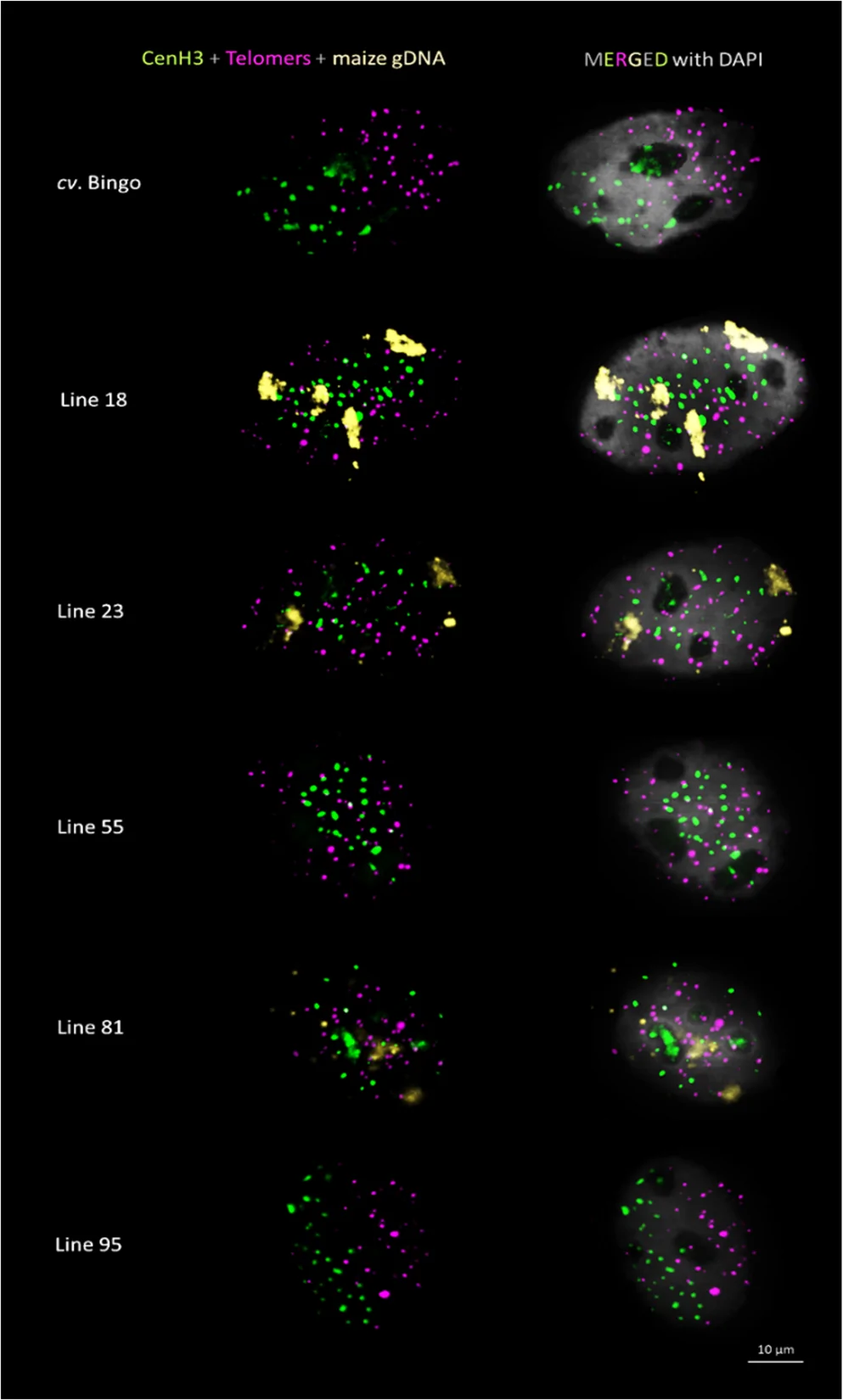
Chromatin configuration in oat *cv*. Bingo and OMA lines nuclei. CenH3 – green fluorescence, indicates the position of the centromeres; Cy5 – pink fluorescence, indicates location of telomeres; Cy3 – yellow fluorescence, indicates location of genomic maize DNA. Chromatin counterstained with DAPI. Scale bar: 10 μm. Photo credit: Katarzyna Juzoń-Sikora

Genomic in situ hybridization (GISH) was used on root-tip metaphase chromosome spreads to determine the number of maize chromosomes added to the oat genome (Fig. 2). All tested lines retained a complete set of oat chromosomes (2n = 42). GISH analysis revealed four added maize chromosomes in line 18 (2n = 46 = 42 + Zm1′′ + Zm2′′) and two added maize chromosomes each in OMA 23 (2n = 44 = 42 + Zm2′′), and OMA 81 (2n = 44 = 42 + Zm5′′) (Juzoń et al. 2020). The presence of *35 S rDNA* loci was detected in oat chromosomes. There was six *35 S rDNA* loci signals as oat is a hexaploid with three subgenomes (A_s_A_s_C_s_C_s_D_s_D_s_ genome) (Jellen et al. 2024). In contrast, lines 55 and 95 showed no detectable GISH signals (Figs. 2 and 3), which was concordant with the lack of Grande-1 maize retrotransposon indicated by polymerase chain reaction (PCR) results (Fig. 1).

### Disturbances of Rabl-like configuration in OMA lines

The polar positioning of centromeres and telomeres in interphase nuclei, referred to as the Rabl configuration, is a characteristic feature of the oat genome but is absent in maize (Nemeckova et al. 2020). In the oat *cv*. Bingo, centromeres and telomeres were consistently located at opposite poles of the nucleus, representing a typical Rabl-like configuration (Fig. 3). In contrast, in the lines containing visible maize chromatin introgression (line 18, 23, and 81), centromeres and telomeres seemed to be scattered throughout the nucleus instead of assuming polarized arrangement. Interestingly, the results of centromere and telomere positioning obtained for lines 95, which lost maize chromatin introgression, indicated the presence of Rabl-like configuration.

In addition, maize chromatin appeared as distinct segments visualized by GISH within the OMA nuclei: four signals were detected in line 18, and two signals in both line 23 and line 81, corresponding to the number of added maize chromosomes in each line. It seems that the maize chromatin formed distinct regions, usually positioned near the nuclear periphery (Fig. 3).

### Maize chromatin introgression can alter cell cycle dynamics and affect genomic stability in OMA lines

**Fig. 4:**
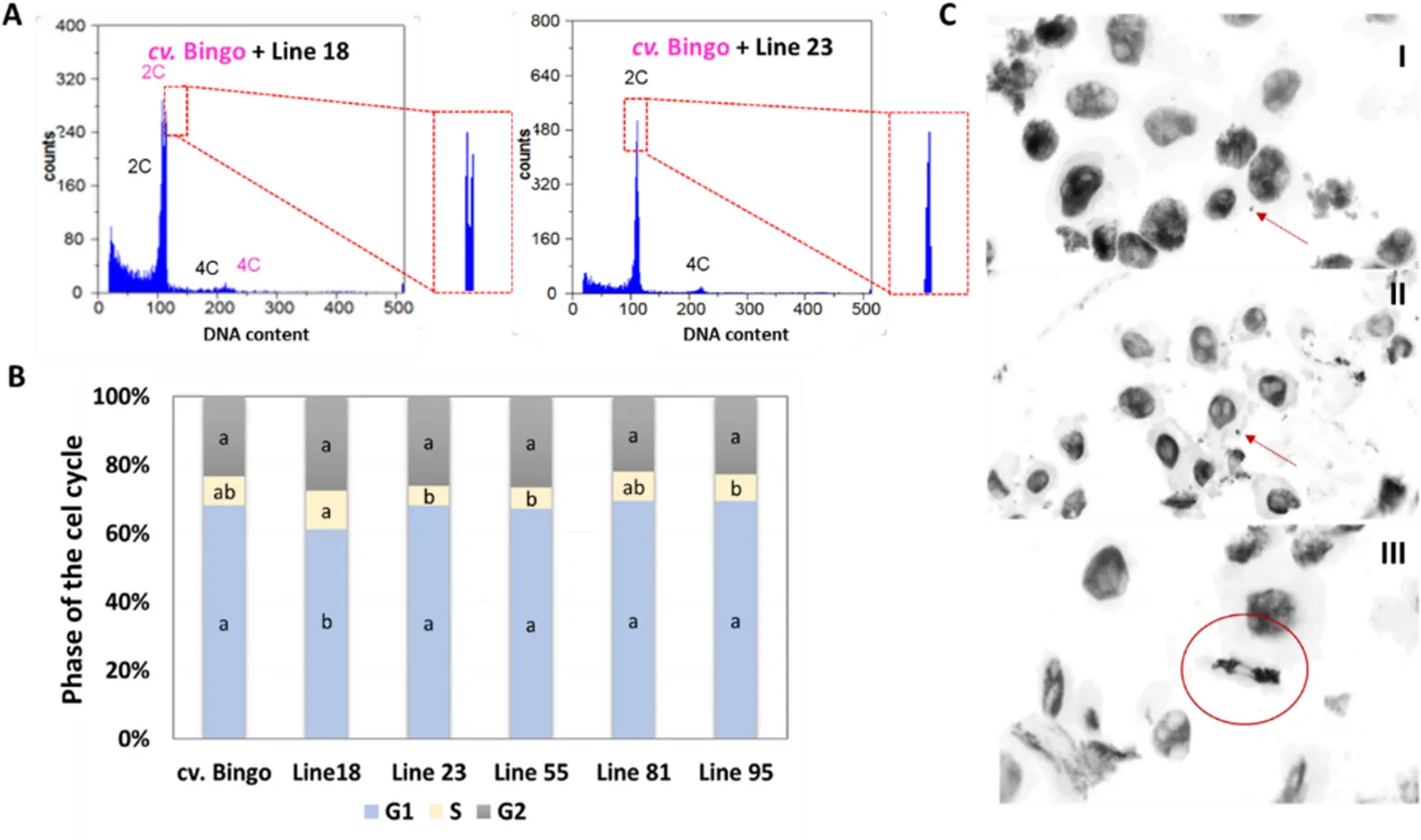
Hybrid cell cycle disorders. (**A**) Histograms of nuclear DNA content obtained from root apical meristem analysis; the X-axis shows the DNA content, the Y-axis the number of measured particles; (**B**) Cell cycle phases of the OMA lines and oat *cv*. Bingo; G1 phase – cell growth; S phase – DNA replication; G2 phase – spindle protein synthesis; ANOVA; *n* = 5; significant differences between the OMA lines according to Duncan’s test, *p* ≤ 0.05, are marked with different letters; (**C**) DNA damage at the level of chromosomes of the line 23: I, II) micronuclei (red arrows); III) anaphase bridge. Photo credit: Katarzyna Juzoń-Sikora

To evaluate the impact of maize chromatin introgression on the nuclear DNA content of tested lines, flow cytometric analyses were conducted. Nuclei from the oat *cv.* Bingo were co-analyzed with those from OMA lines to facilitate direct comparisons. Notably, in line 18, which harbors four additional maize chromosomes, the G₁ (2 C) peak was shifted to higher fluorescence values relative to *cv*. Bingo, indicating an increased nuclear DNA content attributable to the maize chromatin introgression (Fig. 4A). In contrast, line 23 and line 81, each containing a single pair of maize chromosomes, did not exhibit such peak shifts. Subsequent cell cycle phase quantification revealed that line 18 exhibited a modest but consistent increase in the proportion of nuclei in the S phase compared to *cv*. Bingo, implying a prolonged DNA replication phase potentially due to the presence of maize chromosomes (Fig. 4B).

A preliminary assessment of the impact of maize chromatin introgression on genomic stability in OMA lines, was made by Feulgen staining of RAMs to detect mitotic abnormalities. Examples of micronuclei as well as anaphase chromosome bridges were found, particularly in line 23 (Fig. 4C). However, further statistically supported analyses will be necessary to assess their impact on genomic stability.

### Phenotypic impact of maize chromatin introgression on panicle and seed traits in OMA lines

**Fig. 5:**
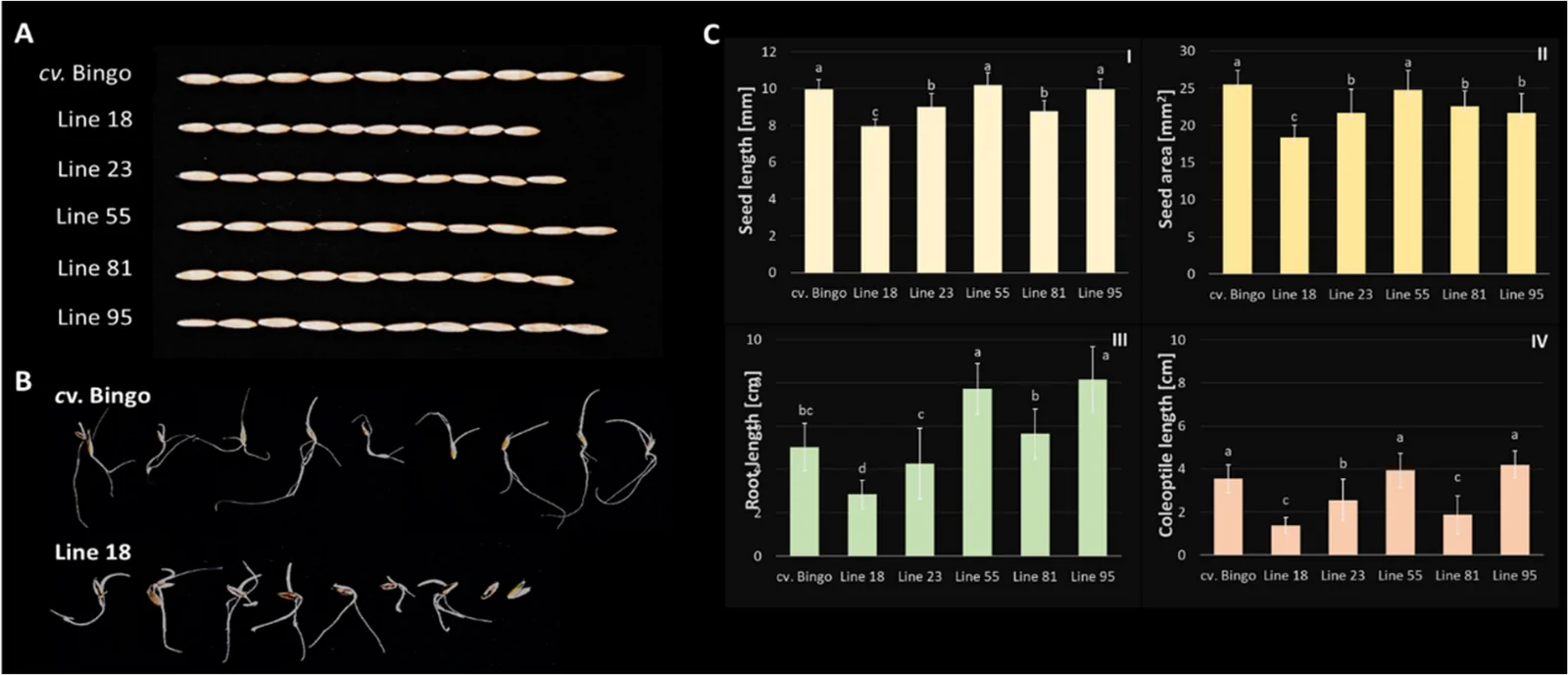
Characteristics of oat and OMA lines seeds and seedlings. (**A**) Seeds of oat *cv*. Bingo and oat lines used in the experiment; (**B**) Comparison of seedlings of oat *cv*. Bingo and line 18. Photo credit: Katarzyna Juzoń-Sikora. (**C**) Seedlings of oat *cv*. Bingo and OMA lines: (I) seed length [mm]; (II) seed area [mm^2^]; (III) root length [cm]; (IV) coleoptile length [cm]; ANOVA; *n* = 10, significant differences between the OMA lines according to Duncan’s test, *p* ≤ 0.05, are marked with different letters

To evaluate the phenotypic consequences of maize chromatin introgression in OMA lines, various panicle and seed traits were analyzed. Seeds of the OMA lines used in the experiment were compared in terms of their size (Fig. 5A). Notable differences were observed between the lines. Oat *cv*. Bingo and line 55 had the longest seeds with the greatest area (Fig. 5C I, II). The seeds of line 95 had a significantly smaller area, even though they were as long as those of oat *cv*. Bingo. Line 23 seeds were of intermediate size. The seeds of the line 18 were the smallest in terms of both length and area. The size of the seeds did not directly correspond with the length of the root during germination (Fig. 5C III). Seedlings’ growth seemed to be affected by maize introgression. Roots of *cv*. Bingo seedlings reached approximately 5 cm in length, whereas roots of line 18 seedlings averaged only 3 cm. Coleoptile growth was also reduced: both line 18 and line 81 seedlings developed coleoptiles shorter than 2 cm, compared to approximately 4 cm in *cv*. Bingo (Fig. 5C III). The longest roots (8 cm) and coleoptiles (4 cm) characterized lines 55 and 95. Germination disorders of hybrid seeds were illustrated in Fig. 5B, showing a comparison between oat *cv*. Bingo and line 18 – shorter roots and/or coleoptiles or their complete absence in some seeds of the OMA line indicated a negative impact of alien genetic material on seed viability.

**Fig. 6:**
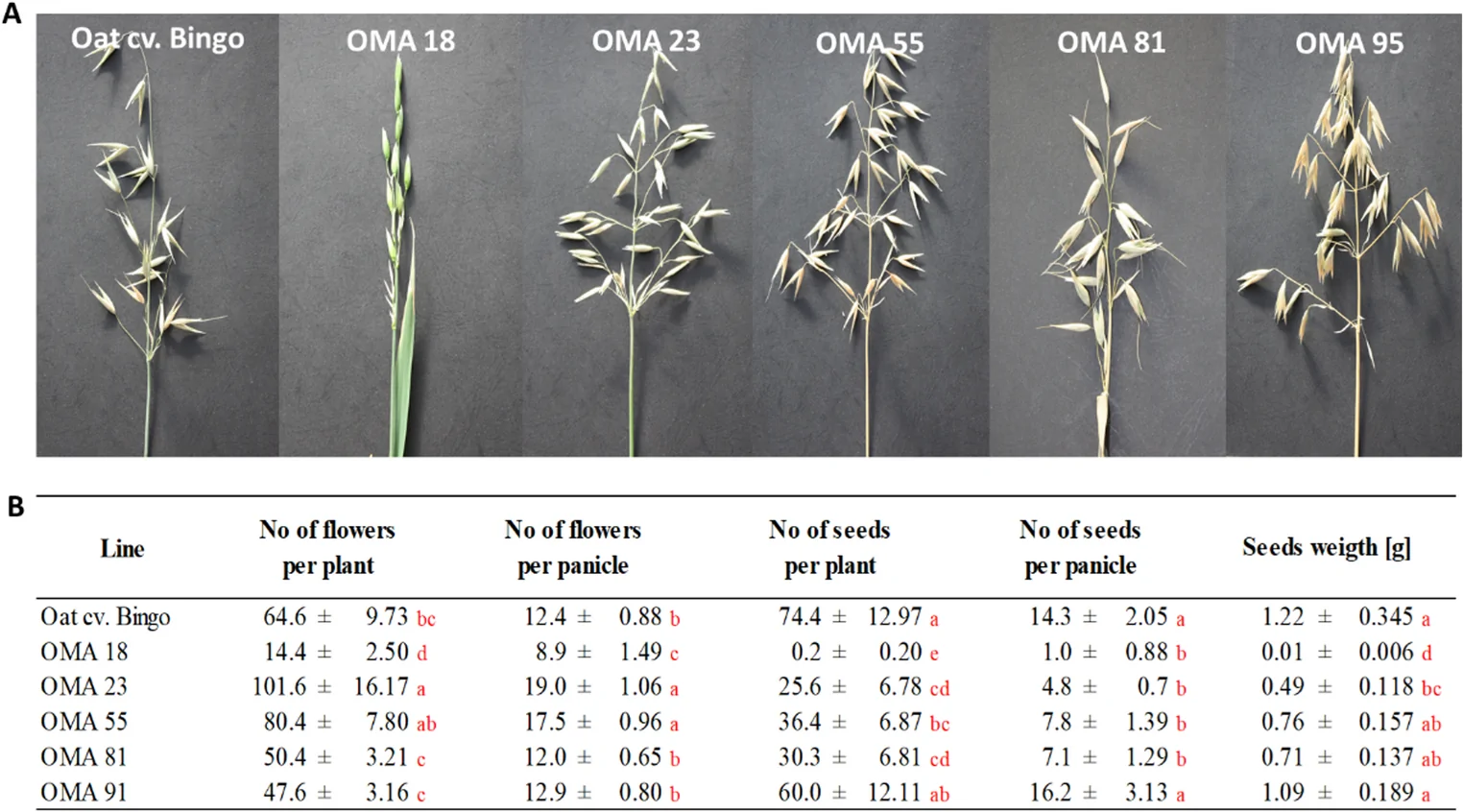
Morphology of oat and OMA plants (**A**) and chosen yield components (**B**): number of flowers per plant, number of flowers per panicle; number of seeds per plant; number of seeds per panicle, seeds weight [g]; ANOVA; *n* = 5; significant differences between the OMA lines according to Duncan’s test, *p* ≤ 0.05, are marked with different letters. Photo credit: Kamila Laskoś

Next, some of the seeds of the tested lines were planted into soil, which enabled conducting further observation of their development. Remarkably, line 18 exhibited a prolonged vegetative phase. While oat *cv*. Bingo plants had completed senescence, by the time of seed harvest, the plants of line 18 remained green, suggesting delayed maturation (Fig. 6A).

Introgression of two maize chromosome pairs into line 18 had a pronounced negative effect on reproductive development. The number of flowers per plant was nearly five-fold lower compared to *cv*. Bingo, and the average seed number was reduced to approximately one seed per plant. In contrast, line 23 showed a positive effect on flower production. The number of flowers per plant and per panicle increased by approximately 1.6-fold compared to *cv*. Bingo. However, this did not translate into higher seed yield, as only one-quarter of the flowers produced viable seeds. Line 81 exhibited a moderate reduction in the number of flowers per plant (approximately 20% lower than *cv*. Bingo), which was accompanied by a substantial (~ 60%) decrease in seed number.

Maize chromatin also impacted seed weight. In line 18, this yield component was reduced by approximately 83% relative to *cv*. Bingo. In lines 23 and 81, a decline by 58% and 42%, was observed, respectively. In line 55 the decrease in weight was 38% compared to *cv*. Bingo. In all tested OMA lines, seeds were visibly smaller compared to the reference cultivar.

## Discussion

Alien chromatin incorporated into crop cultivars can extend the gene pool and make the crops more tolerant to biotic and abiotic stress (Dong et al. 2020). Therefore, the fusion of genomes of unrelated species through wide hybridization is of great significance for plant breeders (Skrzypek et al. 2018). However, the practical value of addition lines depends on the fact that the introgressed chromatin remain stable over generations (Idziak-Helmcke et al. 2020).

Our studies revealed that only 3 out of 5 lines remained OMA lines after three sexual generations. They retained maize chromatin as follows: line 18 with two pairs of chromosomes 1 and 2; line 23 with one pair of chromosomes 2; and line 81 with one pair of chromosomes 5, which is the most frequently retained maize chromosome in oat (Kynast et al. 2001). Therefore, these results are only partially consistent with our previous studies where the younger generation of all tested lines retained maize chromosomes (Juzoń et al. 2020). In single-chromosome wheat-rye addition lines, rye chromosomes pair poorly and were eliminated over time (Orellana et al. 1984). Naranjo (2018) showed that reduced pairing of rye chromosomes in wheat might be due to a lower probability of rye telomeres clustering into the leptotene bouquet at the onset of meiosis.

One of the possible positions assumed by chromosomes in the interphase nucleus is the Rabl-like configuration (Dong and Jiang 1998; Rabl 1885) in which centromeres and telomeres are gathered on opposite sides of the nucleus, resulting in a polarized distribution of interphase chromatin. This polarized orientation is considered to be a direct consequence of an anaphase chromosome arrangement (Cowan et al. 2001; Kaduchová et al. 2023) and seems to be reinforced by mitotic cell divisions (Nowicka et al. 2023).

Oat, with the genome size of ~ 11.3 Gb/1 C, has been previously shown to have Rabl configuration, whereas maize, a moderately-sized genome plant (~ 2.3–2.7 Gb/1 C), was reported to lack such arrangement (Nemeckova et al. 2020). In our study, the result of mapping centromeres and telomeres in oat root tip nuclei was in full concordance with previous research as we observed a polarized configuration of these chromosome domains. In contrast, in the nuclei of OMA lines with added maize chromosomes, this polarized arrangement seemed to be disturbed and a certain degree of dispersion of centromeres and telomeres across the nuclear space was registered. Interestingly, line 95, which carried maize chromatin introgression in the earliest generations but later lost them entirely, apparently displayed the Rabl-like configuration. These observations allow for a cautious assumption that the presence of maize chromosomes may disrupt the polarized organization of the nuclei in OMA lines. Our data are preliminary and would require further, more detailed and quantitative studies on a larger sample of nuclei with a fully preserved 3-D structure. If our hypothesis is confirmed it would raise an interesting question whether the disturbed Rabl-like configuration in OMA lines is merely a consequence of the presence of alien chromatin in oat – a species that normally displays the Rabl-like configuration – or whether the fact that maize is a non-Rabl species plays a significant role. There is very limited data on the nuclear distribution of centromeres and telomeres in hybrids between Rabl-like and non-Rabl-like plants. In the root nuclei of the allotetraploid *Brachypodium hybridum*, the non-Rabl-like configuration is a predominant nuclear organization pattern, whereas its putative parents – *B. distachyon* and *B. stacei* – respectively do and do not exhibit the Rabl-like arrangement in that tissue (Idziak et al. 2015). On the other hand, in various wheat × rye and wheat × barley disomic substitution and addition lines, where both species involved were Rabl-positive plants in their native form, the Rabl-like orientation was also present and highly stable among different tissue types and throughout the cell cycle (Kolackova et al. 2019; Pernickova et al. 2019). The added or substituted chromosomes adopted a string-like configuration in the host nuclei, stretching between the centromere- and telomere-occupied nuclear poles. This remains in striking contrast to the situation we observed in OMA lines, where the added maize chromosomes assume cloud-like shapes in the oat nucleus (see also Idziak-Helmcke et al. 2020). Similar observations were described for the pearl-millet (also a non-Rabl plant) chromosomes in the wheat background (Gernand et al. 2005).

It might therefore be hypothesized that the (still uncharacterized) mechanisms responsible for establishing Rabl orientation in Rabl-positive species may not properly accommodate chromosomes originating from non-Rabl species. The reasons for such incompatibility remain unclear and represent an interesting challenge for future studies. The OMA lines, together with these lines that lost the maize chromatin introgression, provide an excellent system to further investigate these processes.

In our study, we applied the flow cytometry technique to compare the nuclear DNA content between the OMA lines and oat *cv*. Bingo. We found that a double disomic line 18 can be easily distinguished from oat by flow cytometry. We hypothesize the presence of four maize chromosomes as being responsible for the shift in the position of the line 18 G1 peak towards higher nuclear DNA content values. Other studies have similarly reported the identification of chromatin introgression in hybrid nuclei. Bashir et al. (1993) were able to detect rye chromosomes in seven different wheat-rye addition lines, as well as calculate the amount of DNA per rye chromosome. Also, Pfosser et al. (1995) observed changes in nuclear DNA content resulting from the gain or loss of whole chromosomes or chromosome arms in wheat–rye addition lines and in triticale. Using flow cytometry analysis, they identified aneuploidy in nearly all lines studied, except for the one carrying very short telocentric rye chromosomes. In our study, however, flow cytometry failed to distinguish between oat and the single disomic OMA lines 23 and 81, suggesting that the amount of DNA contributed by a single pair of maize chromosomes was insufficient to be detected using this method. In oat (2n = 6x = 42, ~ 11.3 Gb), adding two maize chromosomes (~ 460 Mb, ~ 4% of the host genome) produces a change too small to resolve by flow cytometry, as it is masked by natural peak broadening. Nuclei with four maize chromosomes (~ 920 Mb, ~ 8% of the host genome) could be distinguished, but this represents probably the detection limit. To illustrate when detection is possible, studies by Karafiátová et al. (2021, 2024) and Blavet (2021) on sorghum and maize distinguished nuclei with two or more supernumerary B chromosomes from non-carriers. In sorghum (2n = 2x = 20, ~ 1.5 Gb), nuclei with two B chromosomes (~ 212–280 Mb, 14–19% of the host genome) can be clearly separated from non-carriers. In maize (2n = 2x = 20, ~ 2.3 Gb), nuclei with two B chromosomes (~ 212–280 Mb, 9–12% of the host genome) are near the detection limit and not reliably separable, whereas four B chromosomes (~ 424–560 Mb, 18–24% of the host genome) produce a clearly separable peak. These examples show that the detectability of extra chromosomes depends not only on their proportion relative to the total genome, but also on genome size, as larger genomes produce broader peaks that can mask small shifts, explaining why nuclei with two maize chromosomes in oat cannot be reliably distinguished.

The double disomic OMA line 18 also displayed a higher proportion of S-phase nuclei compared to oat *cv*. Bingo, which may indicate an increase in the duration of DNA replication associated with the higher nuclear DNA content. This is an unexpected result since generally the length of the S-phase has been shown to be rather independent of DNA content in comparisons among various diploid as well as between diploid and polyploid plant species (Mickelson-Young et al. 2016; Troy and Wimber 1968; Verma and Lin 1979).

In the studied OMA lines, we have observed micronuclei formation and mitotic abnormalities including chromosomal bridges in anaphase. These phenomena have been reported multiple times in addition lines derived from wide crosses of the plant species and are related to uniparental chromosome elimination (Gernand et al. 2005; Ishii et al. 2010; Lange 1971; Mochida et al. 2004). However, in such wide crosses, chromosome elimination occurs during the early stages of hybrid embryo development (Gernand et al. 2005; Ishii et al. 2010; Laurie and Bennett 1989). The fact that we found these abnormalities in the cells of the F3 generation of the OMA plants implies that maize chromatin introgression may have long term influence on the genome stability of the OMA lines. This instability could be responsible for losing the added chromosomes in some lines in subsequent generation.

Future experiments should include analyzing whether the mitotic abnormalities specifically involve only maize chromosomes or if oat chromosomes are also affected. Additionally, it would be interesting to quantify the occurrence of such mitosis-related anomalies in OMA lines with different chromatin introgression and compare whether the identity of the added chromosome influences their rates.

In higher plants, the species-specific, complete number of chromosomes present in a nucleus is usually necessary for proper development. The gain or loss of chromosomes causes genomic instability, as normal diploid cells cannot function properly with an imbalance in the chromosomes (Hirano et al. 2014). Therefore, it is expected that maize chromatin introgression can alter multiple developmental and reproductive traits in oat, with the magnitude and direction of effects dependent on the specific maize chromosome(s) introduced.

Our studies revealed some morphological differences within the tested lines. Interestingly, the seeds of lines 55 and 95, which lost maize chromatin, were similar in size to the oat *cv*. Bingo and larger than those of OMA lines. Unfortunately, at the moment we do not have evidence to what extent the differences in seed size, root, and coleoptiles length are related to the oat genotype or rather to the introgression of maize chromatin, and if so, whether the number or type of retained chromosomes is more important. In conducted research oat *cv*. Bingo showed the highest values of seeds number per plant/panicle as well as seeds weight. Similar parameter values were obtained by line 95. In turn, the highest number of flowers per plant/panicle was noted for line 23, which retained two maize chromosomes 2 (single disomic) (Juzoń et al. 2020). Other known OMA lines bearing maize chromosome 2 were characterized by later maturation and had an intense bluish leaf color, with more covered wax stems. Moreover, chromosome 2 addition lines are known to have the highest fertility among all oat-maize addition lines produced to date (Kynast et al. 2001). Line 81, with two retained maize chromosomes 5 (single disomic), produced seeds similar in size, length, and area to the seeds of line 23. Kynast et al. (2001) showed that hybrids with maize chromosome 5 tended to develop fewer but thicker tillers and senesce earlier than the corresponding haploid oat genotypes, but in general, their phenotypes were similar to oat phenotype, which was also observed in our research. In contrast, Warchoł et al. (2024) showed that OMA lines with two maize chromosomes 5 (42 + Zm5”) produced the smallest number of seeds per plant (0.99) among all tested lines.

Compared to the other lines, line 18 with retained two pairs of maize chromosomes 1 and 2 (doubled disomic), exhibited poor viability and delayed maturation. In our previous study, this line manifested in bushy shoots and poor grain yield (Juzoń et al. 2020). According to Riera-Lizarazu et al. (1996) plants with three or four maize chromosomes showed visibly weaker vitality than haploid plants with no maize chromosomes, while hybrids with one or two maize chromosomes looked more similar to oat plants, which was also verified later by the research of Phillips and Rines (2009).

Kynast et al. (2001) indicated that hybrids with chromosome 1 under long-day exposure responded with shortened leaf blades and pollen sterility. These findings were confirmed by our research, where F3 of line 18 had the fewest seeds per panicle. However, the earlier generation (F2) produced a comparable number of seeds as the F3 generation of line 55, which at that time retained maize chromosome 3, making the hybrids the most morphologically distinct. Kynast et al. (2001) showed that the erect leaf phenotype was caused by the presence of maize chromosomes in the oat genome and also found the presence of necrotic and chlorotic spots on the leaf blades compared to the oat *cv*. ‘’Sun II’’. Also Cabral (2009) demonstrated that OMA lines show specific phenotypes, which can indicate that maize genes are expressed in oat. Dong et al. (2018) found that more than 70% of maize genes preserved the original expression or transcription pattern in the oat genome, and a local *cis* region in the maize chromosome may be responsible for that. They postulated also that differences in hybrid morphology may result from the fact that: (i) some maize genes can be expressed in an ectopic way in an oat genomic background; (ii) the interaction between the oat and maize transcriptomes may affect how phenotype-linked oat genes are expressed, and the second mechanism seems to be more possible. The results were also confirmed by Skrzypek et al. (2018), but were contradictory to Warzecha et al. (2023), where all fifteen OMA lines were oat-shaped plants.

## Conclusion

The results suggest the complexity of intergeneric hybrid functioning, which appeared to be influenced more by the specific maize chromosome and its interaction with the oat genome, rather than solely by the number of retained chromosomes. The results suggest that even partial maize chromatin introgression may influence nuclear architecture, including an apparent disturbance of the typical Rabl-like centromere-telomere polarization, cell cycle dynamics, and plant development. The observed cytological abnormalities and lower yield component values may reflect the challenges associated with maintaining genome stability in hybrids and simultaneously underscore the value of OMA lines as experimental objects for studying chromatin maintenance and chromosome organization. Although chromatin introgression can pose challenges to plant development and productivity, it also provides a valuable platform for analyzing the mechanisms of chromosome organization and identifying maize-derived traits of potential breeding significance. Further studies of OMA lines will be essential to assess their long-term stability and to better understand their potential in both basic research and crop improvement.

Our findings also raise new questions in the fields of the mechanism of alien chromatin introgression and its interaction with the host genome, highlighting the need for further research on the formation and functioning of interspecific and intergeneric hybrids.

## Supplementary Information

Below is the link to the electronic supplementary material.

Supplementary Material 1

## Data Availability

The data will be available from corresponding authors on request.

## Abbreviations

CENH3: centromere–specific histone H3
CF: chlorophyll a fluorescence
DH: doubled haploid
FISH: fluorescence in situ hybridization
gDNA: genomic DNA
GFP: green fluorescent protein
GISH: genomic in situ hybridization
OMA: oat × maize addition
PBS: phosphate buffered saline
RAMs: root apical meristems
rDNA: ribosomal DNA
RFP: red fluorescent protein
RT: room temperature
SSC: saline sodium citrate buffer
